# A DEMENTIA IMMERSION SIMULATION EXPERIENCE AS A TRAINING TOOL FOR HEALTH CARE PROFESSIONALS

**DOI:** 10.1093/geroni/igac059.2777

**Published:** 2022-12-20

**Authors:** Sandra Sanchez-Reilly, Paula Reilly, Elizabeth Reilly, Isabella Silva, Lyda Arevalo, Gabriela Zaragoza

**Affiliations:** South Texas Veterans Health Care System and the University of Texas Health Science Center at San Antonio, San Antonio, Texas, United States; South Texas Veterans Health Care System, San Antonio, Texas, United States; South Texas Veterans Health Care System, San Antonio, Texas, United States; South Texas Veterans Health Care System, San Antonio, Texas, United States; South Texas Veterans Health Care System, San Antonio, Texas, United States; UTHSCSA, San Antonio, Texas, United States

## Abstract

**Background:**

Dementia, a progressive, devastating and incurable disease affects millions of older Americans and their caregivers. Effective dementia-training programs for caregivers and healthcare professionals (HP) can lead to improved patient outcomes. Simulation-educational experiences are innovative with long-lasting impact. Currently, there are no simulation hands-on experiences focused on dementia care to train HP or hired caregivers (HC). Objective: To evaluate the educational effectiveness of a Dementia Immersion Simulation Experience (DISE) intervention among HP and HC.

**Methods:**

DISE is a face-to-face 2-hour intervention that includes virtual reality, hands-on simulation with multiple sensory experiences, group debriefing led by dementia-caregiving expert. Program evaluation and pre/post knowledge assessments were administered.

**Results:**

Nf110. HP, Nf72; HC, Nf25. Pre/post mean score of knowledge assessment (scale 0–12) for all was 8.3/9.7 (p < 0.0001). Participants were also grouped by whether they were “well-informed” (achieving 10 or better on knowledge assessment) or had “knowledge gap” (9 or fewer). 25% were well-informed on dementia before DISE; 63% after DISE (p < 0.0001). DISE program evaluation showed 98% participants highly rated experience across all categories. Evaluation scores further support an effective program. Furthermore, number of clinical referrals seeking expertise from a dementia specialist/team increased by 50% two months post-intervention.

**Conclusion:**

DISE is a successful tool to teach, support and empower HP and HC, effectively changing care provided to individuals with dementia by using hands-on simulation training. Further studies are needed to evaluate the effectiveness of DISE in improving behavioral symptoms, training family caregivers, and decreasing other dementia-related undesirable outcomes such as nursing home placement.

